# Prevention of common mental disorders among women in the perinatal period: a critical mixed-methods review and meta-analysis

**DOI:** 10.1017/gmh.2022.17

**Published:** 2022-03-23

**Authors:** Ahmed Waqas, Syeda Wajeeha Zafar, Hafsa Meraj, Mahjabeen Tariq, Sadiq Naveed, Batool Fatima, Neerja Chowdhary, Tarun Dua, Atif Rahman

**Affiliations:** 1 Institute of Population Health, University of Liverpool, Liverpool, UK; 2 Human Development Research Foundation, Islamabad, Pakistan; 3 Institute of Living, Hartford, Connecticut, USA; 4 Department of Mental Health and Substance Use, World Health Organization, Geneva, Switzerland

**Keywords:** Prevention, postpartum depression, postpartum anxiety, meta-analysis, qualitative

## Abstract

Perinatal depression and anxiety account for a high burden of perinatal morbidity and poor psychosocial functioning. There is a growing interest among mental health professionals, to devise interventions to prevent this condition. This review synthesizes evidence for the effectiveness of psychological and psychosocial interventions aimed at the prevention of perinatal depression and anxiety. We also explore qualitative evidence to understand the acceptability and feasibility of these interventions. Using a mixed-methods approach, data from a total of 21 studies were collated to inform the evidence for preventive interventions for perinatal depression and anxiety. Based on their theoretical orientations, these interventions were described by authors as cognitive-behavioral (*n* = 7); psychoeducational (*n* = 6); mindfulness (*n* = 2); and interpersonal psychotherapy (*n* = 2). These also included psychosocial approaches such as social support (*n* = 1) and multicomponent interventions (*n* = 3). For depressive symptoms, these interventions yielded moderate to strong effect sizes in favor of the intervention group [standardized mean difference (SMD) = −0.59; 95% confidence interval (CI) −0.95 to −0.23]. For anxiety symptoms, a strong effect size was estimated in favor of the intervention group (SMD = −1.43, 95% CI −2.22 to −0.65). Preventive interventions significantly reduce the severity of perinatal depressive and anxiety symptoms. These interventions are also acceptable and feasible in many settings.

## Introduction

For many women around the world, pregnancy marks the beginning of a journey toward motherhood. Although pleasant for many, it can trigger the onset of perinatal anxiety and depression, causing distress and disability among pregnant and postpartum women. Although both disorders are recognized as separate clinical entities, they often occur together (Waqas *et al*., 2015) and are referred to as common mental disorders. These disorders pose a global health concern due to their high prevalence and adverse maternal and child consequences. According to Fisher and colleagues, perinatal common mental disorders have a prevalence in low- and middle-income settings of 15.6% [95% confidence interval (CI) 15.4–15.9] during the antenatal period and 19.8% (95% CI 19.5–20.0) in the postpartum period (Fisher *et al*., 2012). In Pakistan, the prevalence of perinatal depression is suggested to be as high as 30% prenatally and 37% during the post-partum period (Atif *et al*., 2021). Despite a high burden of illness, less than 20% of women report their symptoms to healthcare providers due to stigma and poor help-seeking practices inherently associated with these disorders (Muzik *et al*., 2011). Untreated postpartum depression and anxiety have been shown to affect both maternal and child health (Stein *et al*., 2014; Gelaye *et al*., 2016; Waqas *et al*., 2018). In the USA alone, these disorders accounted for a societal loss of 14.2 billion USD in 2017 (Luca *et al*., 2020).

Among pregnant women and mothers, symptoms of anxiety and depression are often associated with a higher risk of comorbid psychiatric conditions such as posttraumatic stress disorder and suicidal behaviors and increased fear of childbirth and thoughts of harming the child (Dikmen-Yildiz *et al*., 2017). These conditions can have a profound impact on the parent–child relationship which is the foundation of the future emotional, relational, and social development of the child (Dubber *et al*., 2014). Untreated perinatal depression and anxiety can put the infant at a higher risk of physical and behavioral ill-health such as preterm births, poor APGAR (appearance, pulse, grimace, activity, and respiration) scores at birth, delayed growth, emotional and behavioral problems, and neurodevelopmental delay (Stein *et al*., 2014; Gelaye *et al*., 2016; Waqas *et al*., 2018). A child born to a mother with depression and/or anxiety lacks the essential ingredients of a nurturing environment, introducing a vicious cycle of inequity, disparity, and intergenerational trauma even before the child is born (Stein *et al*., 2014; Zafar *et al*., 2014; Gelaye *et al*., 2016).

Due to the multilevel impact of perinatal depression and anxiety, it is important to develop and implement carefully designed interventions for their prevention. There is abundant literature demonstrating the effectiveness of psychosocial interventions for the treatment of these disorders (Dennis and Hodnett, 2007; Singla *et al*., 2017). Based on the same theoretical principles, prevention of postpartum depression and anxiety is possible by targeting known biological, psychological, and socioeconomic risk factors, during pregnancy or the early postpartum period (Dennis and Dowswell, 2014; Curry *et al*., 2019). Previous meta-analytic evidence has shown that psychosocial interventions are effective for the prevention of mental health problems during the postpartum period (Dennis and Dowswell, 2014; Curry *et al*., 2019). In their recently published evidence statement, the US Preventive Services Task Force recommended that counseling interventions provide a moderate net benefit in preventing perinatal depression (Curry *et al*., 2019). However, there is limited or mixed evidence in existing literature regarding the effectiveness of these counseling interventions beyond cognitive-behavioral therapy (CBT) and interpersonal therapy (IPT) (Dennis and Dowswell, 2014; Curry *et al*., 2019). It is also noteworthy that most of the clinical recommendations are limited to the scope of perinatal depression, and fewer evidence synthesis efforts have focused on perinatal anxiety (Dennis and Dowswell, 2014; Curry *et al*., 2019). In addition, most of the clinical guidelines do not report the implementation procedures for these psychological and psychosocial interventions. Psychosocial intervention development requires an iterative and dynamic approach, that leverages theoretical frameworks which are then implemented after accounting for feedback from key stakeholders such as pregnant women, mental health professionals, midwives, and other auxiliary services (Chorpita *et al*., 2005; Morrell *et al*., 2009; Kaaya *et al*., 2013). Importantly, successful interventions are tailored to the needs of the population and their social, religious, and cultural norms that sometimes precipitate depression and anxiety (Fisher *et al*., 2012). Therefore, a realist approach is usually required while mapping these psychological interventions, to collate evidence for their effectiveness in real-world settings (Morrell *et al*., 2009).

This mixed-methods review aims to fill previous research gaps outlined above and expands the scope of previous guidelines which have been limited to high-income countries (Dennis and Dowswell, 2014; Howard *et al*., 2014; Curry *et al*., 2019). Besides exploring the meta-analytical evidence base for the preventive interventions, we aim to explore their theoretical underpinnings using distillation and matching frameworks to delineate the active ingredients (Chorpita *et al*., 2005). We also report the implementation characteristics of these interventions and explore qualitative evidence to understand their acceptability and feasibility in real-world settings.

### Research questions

We asked the following question: For perinatal women, do non-pharmacological interventions to prevent perinatal anxiety and depression, compared with active control groups/usual care, improve maternal mental health and infant outcomes.

## Methods

### Search strategy

This review was conducted as per Preferred Reporting Items for Systematic Reviews and Meta-Analyses (PRISMA) recommendations for systematic reviews. Its protocol was registered at PROSPERO International Register for systematic reviews a priori (Ahmed Waqas, 2020). Using a predefined search strategy (Table 1) adapted from a Cochrane review (Dennis and Dowswell, 2014), we searched PubMed, Web of Science (including MEDLINE), CINAHL, Scopus, PsycINFO, Cochrane Central Register of Controlled Trials (CENTRAL), and Global Health Library, in December 2019. This database search was further supplemented by manual searching of bibliography of eligible interventions for their evaluation and implementation studies (irrespective of study design), Cochrane reviews and US Preventive Service Taskforce guideline documents, and the clinical guidelines by The National Institute for Health and Care Excellent in the UK (Dennis and Dowswell, 2014; National Institute for Health and Care Excellence, 2014; Curry *et al*., 2019). The search process was restricted from 2013 to 2019 to include the latest evidence that complements the previous Cochrane review (Dennis and Dowswell, 2014). Studies not available in English language were excluded because of a lack of resources.

**Table 1.** Search strategy

Concept	Keywords
Condition/population	(“perinatal depression”[ti/ab] OR “postpartum depression”[ti/ab] OR “postnatal depression”[ti/ab] OR “postpartum anxiety”[ti/ab] OR “postnatal anxiety”[ti/ab] OR “perinatal anxiety”[ti/ab] OR “Depression, postnatal”[MeSh])
Type of study	(effectiveness[ti/ab] OR trial*[ti/ab] OR “clinical trial”[ti/ab] OR RCT[ti/ab] OR “randomized clinical”[ti/ab] OR implementation OR evaluation[ti/ab] OR “implementation science” OR feasibility[ti/ab] OR “program development”[ti/ab] OR Fidelity[ti/ab] OR appropriateness[ti/ab] OR acceptability[ti/ab] OR adoption[ti/ab] OR sustainability[ti/ab] OR penetration[ti/ab] OR appropriateness[ti/ab])
Interventions	(non-pharmacological OR “psychoeducational” OR “cognitive behavioural therapy” OR “interpersonal psychotherapy” OR “non-directive counselling” OR “psychological debriefing” OR “supportive interactions” OR “tangible assistance” OR “nonspecialist mediated” OR “group therapy” OR “group session*” OR comprehensive OR integrated OR multifaceted OR “multi, component” OR multidimensional OR holistic OR Community)
Maternal outcomes	(“maternal mortality” OR anaemia OR anemia OR “back pain” OR “breast complications” OR fatigue OR tiredness OR exhaustion OR “sleep deprivation” OR “weight retention” OR well-being OR self-esteem OR stress OR anxiety OR depression OR self-harm OR suicide OR “intimate partner violence” OR readmission OR “length of stay” OR “need of medication” OR “Maternal functioning” OR “emotional attachment” OR selfefficacy, OR competence OR autonomy OR confidence OR selfcare OR “coping skills” OR “infant care” OR “mother-child interactions” OR “daily living” OR “social support” OR “quality of life” OR “responsive care giving” OR cost-effectiveness OR “neonatal mortality” OR infection OR sepsis OR omphalitis OR jaundice OR disability OR allergy OR surgery OR injury OR immunization OR growth OR height OR weight OR “head circumference” OR “motor development” OR “developmental milestone*” OR breastfeeding)

### Inclusion and exclusion criteria

#### Participants/population

- Studies on preventative psychosocial or psychological interventions among perinatal women were considered.
- Only those studies were included that reported maternal anxiety or depression as a primary outcome.
- The populations studied were pregnant women and postpartum mothers, including those with no known risk and those identified as at-risk of developing perinatal depression (pregnant adolescents, women with prodromal symptoms of depression and anxiety, new adolescent mothers, pregnant women in humanitarian settings, single pregnant women, etc.)
- Studies that provided an intervention during the antenatal and postpartum periods were included.
- Trials where more than 20% of participants fulfilled the clinical criteria for depressive disorder at trial entry were excluded; to avoid inclusion of treatment interventions.
- Interventions conducted among perinatal women with medical comorbidities such as hypertension or gestational diabetes mellitus were excluded.

#### Interventions

- Studies that assessed the effectiveness of non-pharmacological (psychosocial and psychological) interventions were included. These included psycho-educational strategies, CBT, interpersonal psychotherapy, non-directive counseling, supportive interactions, non-specialist mediated therapies, and group therapies.
- Multi-dimensional and multicomponent interventions involving psychotherapeutic elements were included.

#### Study design

- *Quantitative evidence*: for quantitative evidence of preventative interventions, we only considered randomized or cluster randomized controlled trials (RCTs).
- *Qualitative evidence and process outcomes*: any evaluation studies (irrespective of study design) of the eligible RCTs exploring our PICO questions were included to understand the implementation processes, acceptability, and feasibility of the interventions.
- Short formats of publications such as brief reports, letters to editors, conference papers, and abstracts were excluded.

#### Outcomes

In line with our primary aim of delineating the effectiveness of interventions in the prevention of perinatal anxiety and depressive disorders, the following primary outcomes were considered:

- Severity of perinatal depressive and anxiety symptoms assessed with psychometric screening scales.
- Rate of perinatal depressive and anxiety disorders according to Diagnostic Statistical Manual (DSM) or International Criteria for Diagnoses (ICD) (World Health Organization, 1992; American Psychiatric Association, 2013).

In addition, we considered several secondary outcomes to assess the effectiveness of the interventions in improving the overall biopsychosocial health of postpartum women and their children. For this purpose, the following outcomes were considered:

- Maternal physical health parameters included rates of maternal mortality and rates of short-term maternal morbidity (anemia, back pain, breast complications, fatigue/tiredness/exhaustion, sleep deprivation, and weight retention).
- Maternal psychological status assessed using validated psychometric scales for assessment of wellbeing, self-esteem, stress, intimate partner violence, suicide, and self-harm.
- Indicators of maternal functioning measured using validated psychometric scales for assessment of emotional attachment, self-efficacy, competence, autonomy, confidence, self-care, and coping skills.
- Pattern of health services use measured as readmission to hospital, length of stay, unscheduled use of health services, and need of medication.
- Infant care measured using psychometric scales for constructs including mother–child interactions and postpartum attachment.
- Post-intervention rates of exclusive and continuous breastfeeding.
- Scores on psychometric measures of daily functioning and perceived social support.
- Quality of life measured using validated scales such as the WHO Quality of Life scale.
- Child health outcomes included post-intervention rates of neonatal mortality, rates of poor health indicators such as infectious illnesses, jaundice, disability, allergy, surgery, injury, and immunization status.
- Parameters of child growth such as height, weight, and head circumference.
- Motor development, developmental milestones, speech, and language development assessed using validated scales such as the Bayley Scales of Infant and Toddler Development.
- *Implementation processes*: acceptability, evaluation, cost-effectiveness, and uptake assessed using qualitative interviews of intervention recipients and delivery agents.
- *Cost*: out-of-pocket expenditures and cost-effectiveness

### Data extraction (selection and coding)

Two reviewers (SWZ, HM, SN, and MT) working independently from one another scrutinized titles and abstracts as per pre-defined inclusion and exclusion criteria. This phase was aided by the use of Rayyan software (Ouzzani *et al*., 2016). Any differences in decisions of these two reviewers were resolved by a senior author (AW). This phase was followed by scrutinizing full texts of studies found eligible in the previous phase. The two reviewers were then trained in the data extraction procedure, where their inter-rater reliability was assessed on 10% of included studies. After establishing good inter-rater reliability, data extraction was performed for the rest of the studies against several matrices including characteristics of publications and study population: theoretical underpinnings and implementation characteristics of interventions.

Characteristics of populations included the age range of mothers and children and criteria for inclusion in trials. Publication characteristics included country and region of study and primary outcomes. Implementation characteristics of these interventions included the setting of intervention, delivery agents, methods for rating competency, and fidelity for intervention delivery. Thereafter, interventions were grouped according to their theoretical underpinnings such as CBT, interpersonal psychotherapy, mindfulness, music therapy, or social support interventions. The therapeutic elements in these interventions were further scrutinized at a granular level using the framework of distillation and matching which propounds those different psychological interventions may have similarities across the range of therapeutic elements it comprises (Chorpita *et al*., 2005), despite following a different taxonomy. Taxonomy for these elements was adapted from a previous systematic review (Singla *et al*., 2017). Qualitative evaluation outcomes of interest were perspectives of patients, researchers, and stakeholders on acceptability and feasibility of these interventions.

### Statistical analysis

All statistical analyses were conducted in Comprehensive Meta-analysis Software (Version 3, New Jersey, USA). For quantitative outcomes, data for both the primary and secondary outcomes were recorded as post-intervention mean (s.d.) and sample size of intervention and control groups. For dichotomous outcomes, we considered the number of events and sample sizes of intervention and control conditions (Higgins, 2019). Study-wise weighted effect sizes and pooled effects sizes for all outcomes were presented as a forest plot. Data pertaining to specific outcomes were pooled using random-effects (DerSimonian and Laird method), because of expected clinical and methodological heterogeneity across the studies (Higgins, 2019). Heterogeneity was considered significant at >40%. Sensitivity analyses using the single-study knockout approach were used to assess the contribution of single studies to specific outcomes. Publication bias was assessed using Begg's funnel plot and Egger's regression statistic (significant at *p* < 0.10), for each outcome reported in 10 or more studies. In case of significant publication bias, the trim and fill method proposed by Duval and Tweedie was to improve the symmetry of the funnel plot, thus, adjusting the pooled effects size for publication bias. Subgroup analyses were run for theoretical orientation of psychological interventions, type of delivery agent, and type of population (general *v.* at risk). A series of meta-regression analyses were conducted to assess the association of dose density of therapy assessed using the number of sessions, duration of each session, and duration of the overall intervention program. Meta-regression was only run when each covariate was reported in more than 10 studies (Borenstein, 2021). Data on the cost-effectiveness of reviewed interventions could not be meta-analyzed due to heterogeneity in reporting of outcomes across these trials.

### Risk of bias (quality) assessment and quality of evidence

The risk of bias among RCTs was assessed using the Cochrane tool for risk of bias assessments (version 1) across five domains: selection bias, performance bias, detection bias, attrition bias, and reporting bias (Higgins, 2019). The risk of bias across these domains was categorized as low, high, and unclear. These domains were classified as being unclear when methodological details provided by the authors were either missing or insufficient. Thereafter, GRADE evidence criteria were used to grade the quality of evidence for these interventions for critical outcomes. The quality of evidence was graded from very low to high based on several criteria including study design, risk of bias, indirectness, imprecision, inconsistency, publication bias, and dose–response relationship (Guyatt, 2011).

### Qualitative data synthesis

For studies reporting the acceptability and feasibility of psychosocial interventions for perinatal depression and anxiety, we adopted a narrative synthesis approach. In this phase, two reviewers working independently from one another rigorously reviewed these studies and extracted relevant quantitative or qualitative (interviews) data. One senior reviewer utilized an open-coding approach to label and categorize the quantitative and qualitative data into broad themes. The number of studies reporting broader themes was quantified and meaningful relationships and inferences were drawn from them.

## Results

**Fig. 1.**
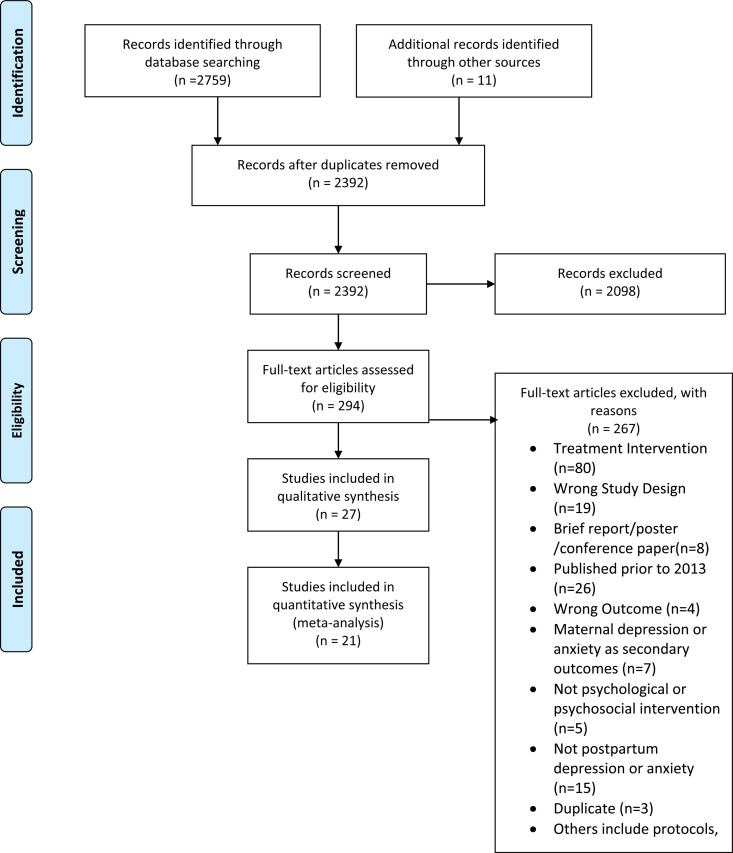
PRISMA flowchart exhibiting study selection process.

The database search process yielded a total of 2659 bibliographic records and 11 articles were added using the manual search method outlined above. After excluding 2098 studies during the title and abstract screening process, we included 294 studies that were further scrutinized during the full-text screening phase (Fig. 1). In the full-text screening process, we excluded 267 studies that were treatment interventions (*n* = 80) or other types of publications such as protocols, studies in languages other than English, and those missing full texts (*n* = 100). After the screening process, a total of 27 studies were included: RCTs (*n* = 21); studies reporting acceptability/feasibility of interventions (*n* = 6); and cost-effectiveness (*n* = 2).

### Description of studies and participants

Data from a total of 21 studies were collated to inform the meta-analytic evidence for preventive interventions for perinatal depression and anxiety. Out of these 21 studies, there were 12 powered RCTs, six pilot RCTs, two quasi-experimental studies, and one cluster RCT. Only two of the trials aimed to test effectiveness of psychological interventions in a pragmatic real-world setting (Fisher *et al*., 2016; Kenyon *et al*., 2016), while the rest were conducted in research settings. Most of these trials were conducted in high-income countries including the USA (*n* = 8), the UK (*n* = 4), and one each in Spain and France, Portugal, Denmark, and Australia. Among middle-income countries, these interventions were only tested in Iran (*n* = 3) and China (*n* = 1). One of the interventions was conducted using an online platform among Spanish and English-speaking pregnant mothers residing in multiple countries (Chile, Spain, Argentina, Mexico, Colombia, and the USA) (Barrera *et al*., 2015). Only 12 of these studies had cited a priori registration of study protocols.

The mean age of intervention recipients across studies ranged from 21 to 40 years. Only one of the studies reported findings among adolescent mothers in the USA (aged 13–18 years) (Phipps *et al*., 2013). Geographically, six of these trials were conducted in urban settings, online (*n* = 5) (Barrera *et al*., 2015; Hantsoo *et al*., 2018; Krusche *et al*., 2018; Duffecy *et al*., 2019; Fonseca *et al*., 2019), multiple settings (*n* = 2) (Brugha *et al*., 2016), and rural (*n* = 1) (Jesse *et al*., 2015). All of the interventions included in this trial were preventative; with either a universal focus (*n* = 10) (Gu *et al*., 2013; Howell *et al*., 2014; Barrera *et al*., 2015; Fathi-Ashtiani *et al*., 2015; Brugha *et al*., 2016; Fisher *et al*., 2016; Krusche *et al*., 2018; Sanaati *et al*., 2018) or a targeted/at-risk focus (*n* = 11) (Zlotnick *et al*., 2006; Phipps *et al*., 2013; Cooper *et al*., 2015; Dimidjian *et al*., 2015; Howell *et al*., 2014; Ortiz Collado *et al*., 2014; Maimburg and Væth, 2015; Kenyon *et al*., 2016; Hantsoo *et al*., 2018; Duffecy *et al*., 2019; Fonseca *et al*., 2019). Interventions with a target focus provided prevention therapies for women on public assistance (*n* = 1), primiparous mothers (*n* = 2), and adolescent mothers (*n* = 1). A majority of interventions targeted postpartum depression (*n* = 18); three trials focused on both anxiety and depression (Fisher *et al*., 2016; Krusche *et al*., 2018; Sanaati *et al*., 2018) and one only on anxiety (Gu *et al*., 2013). Detailed characteristics of the studies are presented in online Supplementary Table S1.

### Characteristics of interventions

**Fig. 2.**
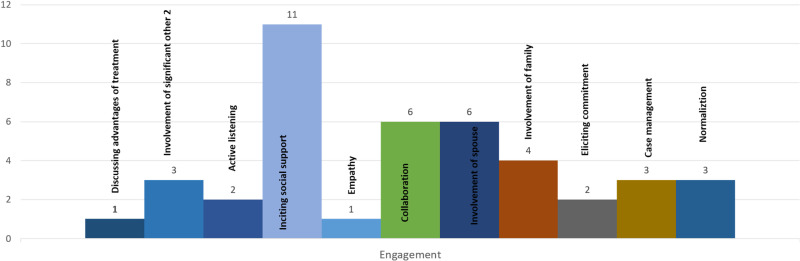
Non-specific elements of interventions.

A total of 15 interventions comprised of elements specific to the psychological or psychosocial domain while six interventions comprised of elements commonly used as in-session techniques (Fig. 2) (Gu *et al*., 2013; Moshki *et al*., 2014; Fisher *et al*., 2016; Kenyon *et al*., 2016; Hantsoo *et al*., 2018; Sanaati *et al*., 2018). The latter group of interventions comprised primarily of psychoeducational modules; however, some of these also provided lay social support (Kenyon *et al*., 2016), mood tracking and alert through software (Hantsoo *et al*., 2018), and midwives run antenatal clinical support (Gu *et al*., 2013). The interventions were delivered either by mental health professionals or lay professionals. Interventions mediated by lay professionals included midwives (Gu *et al*., 2013; Maimburg and Væth, 2015; Brugha *et al*., 2016); health visitors (Cooper *et al*., 2015); facilitators (Phipps *et al*., 2013); pregnancy outreach workers (Kenyon *et al*., 2016), and multidisciplinary teams of nurses, midwives, and graduates (Zlotnick *et al*., 2006; Ortiz Collado *et al*., 2014). Professionals with mental health background delivering these interventions were social workers (Howell *et al*., 2014; Jesse *et al*., 2015); clinical psychologists (Dimidjian *et al*., 2015); mental health nurses (Fisher *et al*., 2016); mental health researchers (Moshki *et al*., 2014); multidisciplinary teams of reproductive health and mental health nurses (Sanaati *et al*., 2018), and licensed social workers, clinical and health psychologists (Fathi-Ashtiani *et al*., 2015). Five of the interventions were delivered through self-help apps or online media (Barrera *et al*., 2015; Hantsoo *et al*., 2018; Krusche *et al*., 2018; Duffecy *et al*., 2019; Fonseca *et al*., 2019). A total of nine interventions were integrated into healthcare settings (Dimidjian *et al*., 2015; Howell *et al*., 2014; Fathi-Ashtiani *et al*., 2015; Jesse *et al*., 2015; Maimburg and Væth, 2015; Brugha *et al*., 2016; Fisher *et al*., 2016; Kenyon *et al*., 2016; Hantsoo *et al*., 2018). Detailed characteristics of the included studies are presented in online Supplementary Tables S1 and S2.

### Therapeutic ingredients of interventions

**Fig. 3.**
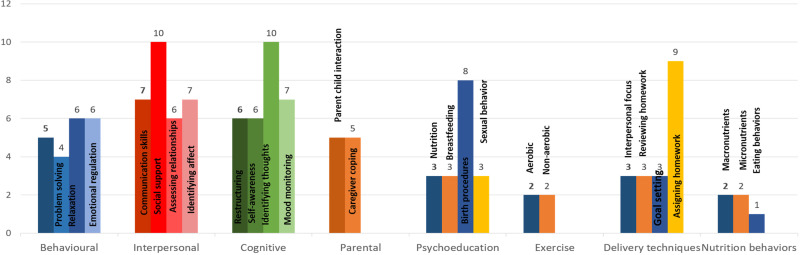
Specific therapeutic elements of interventions.

Based on their theoretical orientations, these interventions were described by authors as cognitive-behavioral (*n* = 7); psychoeducational (*n* = 6); mindfulness (*n* = 2); social support (*n* = 1); interpersonal psychotherapy (*n* = 2); and multicomponent interventions with predominant non-specific therapeutic elements (*n* = 3). Only a few of these interventions were formally manualized, which was a major barrier in identifying the active therapeutic elements based on the distillation and matching model (Chorpita *et al*., 2005). Using the taxonomy proposed by Singla *et al*. (2017), an overlap in therapeutic elements (online Supplementary Table S2; Table 2) across different interventions was identified (Figs 3 and 2).

**Table 2.** Delivery agent and duration of interventions

Author/year	Intervention category	Intervention focus	Population focus	Type of components	Delivery agent of intervention	Integration into healthcare setting	Technology for delivery	Number of sessions of treatment	Duration of one session of treatment	Duration of overall program (weeks)	Booster sessions
Barrera *et al*. (2015)	CBT	Prevention	Universal	Specific	Self-Help app	No	Online	8	Not reported	Not reported	Not reported
Brugha *et al*. (2016)	CBT	Prevention	Universal	Non-specific	Midwife	Yes	Face to face	3	Not reported	Not reported	None
Duffecy *et al*. (2019)	CBT	Prevention	At risk	Specific	Online	No	Online	16	10–15 min	8	3
Fisher *et al*. (2016)	Psychoeducation	Prevention	Universal	Non-specific	Maternal and child nurses	Yes	Online, face to face	1	6 h	One session	0
Fonseca *et al*. (2019)	CBT, acceptance, and compassion based therapy	Prevention	At risk	Specific	Online	No	Online	5	Not reported	5	Not reported
Hantsoo *et al*. (2018)	Psychoeducation/mood monitoring	Prevention	At risk	Non-specific	Online	Yes	Online	Not reported	Not reported	8	Not reported
Howell *et al*. (2014)	Behavioral education	Prevention	At risk	Specific	Social workers	Yes	Face to face	2	15 min in-hospital review + phone call	2	None
Jesse *et al*. (2015)	CBT	Prevention	At risk	Specific	Licensed clinical social worker and other licensed mental health professionals	Yes	Face to face	6	120 min	6	None
Kenyon *et al*. ( 2016)	Not reported	Prevention and promotion	At risk	Non-specific	Pregnancy outreach workers	Yes	Face to face	Not reported	Not reported	Not reported	Not reported
Krusche *et al*. (2018)	Mindfulness	Prevention	Universal	Specific	Online	Standalone intervention	Online	10	Not reported	4	None reported
Dimidjian *et al*. (2015)	Mindfulness-based cognitive therapy	Prevention	At risk	Specific	Clinical psychologists, behavioral health sciences	Yes	Face to face	8	120 min	8	Optional once a month
Tandon *et al*. (2014)	CBT	Prevention	Universal	Specific	Licensed clinical social workers or clinical psychologists	No	Face to face	6	120 min	6	Booster sessions at 3-months and 6-months post-intervention
Sanaati *et al*. (2018)	Lifestyle education	Prevention	Universal	Non-specific	First author of this manuscript (Ph.D. student in Reproductive Health) and for fathers by a person who has M.Sc. degree in the Psychiatric Nursing	No	Face to face	4	60–90 min	24–28	None
Fathi-Asthiani *et al*. (2015)	CBT	Prevention	Universal	Specific	A multidisciplinary team of therapists with a variety of professional backgrounds including midwives, health psychology, psychotics, and licensed clinical psychologist	Yes	Face to face	8	40–60	2	None
Gu *et al*. (2013)	Antenatal education	Prevention	Universal	Non-specific	Midwives	No	Face to face	7	Not reported	Not reported	None
Moshki *et al*. (2014)	Education-based	Prevention	Universal	Non-specific	Not reported	No	Face to face	9	Not reported	36 h	None
Maimburg *et al*. (2015)	Antenatal education	Prevention	Targeted	Specific	Midwives	Yes	Face to face	3	180 min	3	None
Ortiz Collado *et al*. (2014)	Humanist	Prevention	At risk	Specific	Nurses and midwives	NO	Face to face	10	2 h and 15 min		10 weeks
Zlotnick *et al*. ( 2006)	IPT-based	Prevention	Targeted	Specific	Nurse and two individuals with BS degree	NO	Face to face	4	90 min	4	2
Phipps *et al*. (2013)	IPT-based	Prevention	Targeted	Specific	Facilitators	NO	Face to face	5	30–60 min	5	1
Cooper *et al*. (2015)	Supportive therapy, infant behavioral assessment based	Prevention	Targeted	Specific	HS employed health visitors	No	Face to face	11	Not reported	Not reported	Not reported

Overall, the most frequently employed non-specific elements were eliciting social support (*n* = 11), spousal support (*n* = 6), collaboration in care (*n* = 6), involvement of family (*n* = 4), case management (*n* = 3), normalization (*n* = 3), and active listening (*n* = 2) (online Supplementary Figs S1 and S2). The most frequently employed specific elements (online Supplementary Fig. S2) belonged to interpersonal skill categories such as training in assertiveness (*n* = 10) and communication skills (*n* = 7); identifying affect (*n* = 7) and assessment of relationships (*n* = 6) and cognitive skills such as identifying thoughts (*n* = 10), mood monitoring (*n* = 7), self-awareness (*n* = 6), and cognitive restructuring (*n* = 6). Most common behavioral therapeutic elements were relaxation (*n* = 6), emotional regulation (*n* = 6), stress management (*n* = 5), and self-monitoring (*n* = 4). Parenting skills included parent–child interaction (*n* = 5) and parental coping (*n* = 5). Psychoeducational interventions frequently focused on birth procedures (*n* = 8) and breastfeeding, nutrition, and sexual behaviors (each *n* = 3). While specific delivery techniques included assigning homework (*n* = 9), reviewing homework (*n* = 3), and goal setting (*n* = 3).

### Meta-analysis for maternal outcomes

Forest plots presenting the effectiveness of eligible interventions for a variety of outcomes have been provided as online Supplementary Figs S1–S6.

#### Severity of depression symptoms

This outcome was reported in 12 studies (14 data points) among 1864 study participants, where most of the studies employed the Edinburgh Postnatal Depression Scale (EPDS) for measurement of perinatal depressive symptoms (*n* = 10), Patient Health Questionnaire-9 items (*n* = 2), and other (*n* = 2). There was evidence for substantial heterogeneity (*I*^2^ = 92.30%; *Q* = 168.83; *p* < 0.001). These interventions yielded moderate to strong effect size in reducing the severity of depressive symptoms [standardized mean difference (SMD) = −0.59; 95% CI −0.95 to −0.23]. There was no evidence of publication bias in reporting of this outcome on visualization of funnel plot (Egger's regression *p* = 0.30, online Supplementary Fig. S9). Sensitivity analyses did not reveal any significant changes in effect sizes after the removal of individual studies from the pooled analyses.

Subgroup analyses (Table 3) revealed that general populations showed a greater reduction in severity of depressive symptoms (SMD = −0.67, 95% CI −1.29 to −0.06) followed by at-risk populations (SMD = − 0.50, 95% CI −0.89 to −0.10). These differences were statistically non-significant (*Q* = 0.23, *p* = 0.63). Interventions delivered by specialists yielded stronger effect sizes than non-specialists, online apps, or multidisciplinary teams (*Q* = 9.48, *p* = 0.02). Albeit statistically insignificant, CBT-based therapies yielded highest effect sizes (SMD = −0.86, 95% CI −1.56 to −0.17) followed by psychoeducational interventions (SMD = −0.67, 95% CI −1.41 to 0.07). No significant differences in effect sizes were observed among studies employing specific or non-specific intervention elements; or integrated/non-integrated interventions; and mode of delivery or booster dose. Complete information regarding dosage density of interventions was reported in only seven studies. Meta-regression analysis did not reveal any association of number of sessions (*R*^2^ = 0.06, *p* = 0.62), their duration (*R*^2^ = 0.06, *p* = 0.59), and duration of overall programs (*R*^2^ = 0.28, *p* = 0.20) (online Supplementary Figs S7 and S8).

**Table 3.** Subgroup analysis to identify moderators of preventive interventions for perinatal depressive symptoms

Group	Number of studies	Point estimate	Standard error	Variance	Lower limit	Upper limit	*Z*-value	*p* value	*Q*-value	df (*Q*)	*p* value
Time period											
Antenatal	8	−0.89	0.22	0.05	−1.32	−0.46	−0.46	<0.001	4.70	2	0.09
Both	4	−0.27	0.32	0.10	−0.89	0.36	0.36	0.40			
Postpartum	2	−0.01	0.43	0.18	−0.85	0.83	0.83	0.99			
Population focus											
At risk	8	−0.50	0.20	0.04	−0.89	−0.10	−2.48	0.01	0.23	1.00	0.63
Universal	6	−0.67	0.31	0.10	−1.29	−0.06	−2.14	0.03			
Intervention elements											
Non-specific	3	−0.67	0.38	0.14	−1.41	0.07	−1.77	0.08	0.05	1.00	0.82
Specific	11	−0.57	0.23	0.05	−1.02	−0.12	−2.48	0.01			
Delivery agents											
Multidisciplinary	1	0.11	0.17	0.03	−0.23	0.44	0.62	0.54	9.48	3.00	0.02
Non-specialist	4	−0.50	0.44	0.20	−1.37	0.37	−1.13	0.26			
Online	2	−0.44	0.50	0.25	−1.41	0.54	−0.88	0.38			
Specialist	7	−0.78	0.24	0.06	−1.26	−0.30	−3.21	0.00			
Integration in healthcare setting											
No	8	−0.43	0.18	0.03	−0.78	−0.08	−2.40	0.02	0.81	1.00	0.37
Yes	6	−0.81	0.39	0.15	−1.57	−0.05	−2.10	0.04			
Mode of delivery											
Face to face	12	−0.61	0.20	0.04	−1.00	−0.22	−3.07	0.00	0.11	1.00	0.74
Online	2	−0.44	0.50	0.25	−1.41	0.54	−0.88	0.38			
Strategies											
CBT	7	−0.86	0.35	0.13	−1.56	−0.17	−2.43	0.02	6.53	3.00	0.09
Mindfulness	1	−0.24	0.27	0.07	−0.78	0.29	−0.88	0.38			
Other	3	−0.07	0.10	0.01	−0.27	0.12	−0.75	0.45			
Psychoeducation	3	−0.67	0.38	0.14	−1.41	0.07	−1.77	0.08			

#### Rates of depressive disorder

This was reported in six studies (seven trials), conducted among 1003 participants. A higher proportion of these studies employed the SCID-based interviews (*n* = 3). There was some evidence of heterogeneity in reporting of this outcome (49.90%, *p* = 0.08). It revealed a non-significant reduction in rates of depression among intervention recipients (*R*^2^ = 0.86, 95% CI 0.65–1.32). Sensitivity analyses did not reveal any significant changes in effects size for this outcome. No subgroup analyses were conducted for this outcome since few studies reported it.

#### Severity of anxiety symptoms

This outcome was reported in only three studies conducted among 432 participants. All of these studies utilized State-Trait Anxiety Inventory. There was substantial evidence of heterogeneity in reporting of this outcome (*I*^2^ = 92.51%, *Q* = 25.48, *p* < 0.001). Overall, these interventions were associated with a significant reduction in the severity of symptoms of anxiety (SMD = −1.43, 95% CI −2.22 to −0.65). Sensitivity analyses did not reveal any significant changes in effects size for this outcome. No subgroup analyses were run for this outcome due to few studies reporting this outcome.

#### Rates of generalized anxiety disorder

This outcome was reported in only two studies among 499 participants, using GAD-7 and Beck Anxiety Inventory. There was significant evidence of heterogeneity (*I*^2^ = 50.51%, *Q* = 2.02, *p* = 0.12), with no improvement in GAD symptoms [odds ratio (OR) 1.22, 95% CI 0.74–2.01]. Sensitivity analyses did not reveal any significant changes in effects size for this outcome. No subgroup analyses were run for this outcome due to few studies reporting this outcome.

#### Marital problems

This outcome was reported in four studies (six data points) among 1285 participants using varying instruments and subjective questions. There was significant evidence of heterogeneity in the reporting of this outcome (*I*^2^ = 61.21%, *Q* = 12.89, *p* = 0.02). It showed a small effect size in the improvement of marital problems (SMD: −0.23, 95% CI −0.42 to −0.03). Sensitivity analyses did not reveal any significant changes in effects size for this outcome. No subgroup analyses were run for this outcome due to few studies reporting this outcome.

#### Treatment seeking practices

This outcome was reported in only two studies among 980 participants (Kenyon *et al*., 2016; Hantsoo *et al*., 2018). There was no evidence of heterogeneity in reporting of these outcomes. These interventions did not reveal any benefits toward the intervention group in improving treatment seeking practices. Sensitivity analyses did not reveal any significant changes in effects size for this outcome. No subgroup analyses were run for this outcome due to few studies reporting this outcome.

#### Self-esteem

Only two studies reported this outcome among 282 participants. There was substantial heterogeneity in reporting of this outcome (*I*^2^ = 77.55%, *Q* = 4.54, *p* = 0.04). These interventions did not reveal any improvement in self-esteem among intervention recipients (SMD = −0.01, 95% CI −0.50 to 0.49). Sensitivity analyses did not reveal any significant changes in effects size for this outcome. No subgroup analyses were run for this outcome due to few studies reporting this outcome.

#### Satisfaction with treatment

Only two studies reported this outcome among 317 participants. There was substantial heterogeneity in reporting of this outcome (*I*^2^ = 90.72%, *Q* = 10.77, *p* = 0.001). These interventions did not reveal any improvement in satisfaction among intervention recipients (SMD = 0.26, 95% CI −0.66 to 1.88). Sensitivity analyses did not reveal any significant changes in effects size for this outcome. No subgroup analyses were run for this outcome due to few studies reporting this outcome.

#### Maternal morbidity

Only two studies reported the outcome of postpartum hemorrhage among 1339 participants. There was substantial heterogeneity in reporting of this outcome (*I*^2^ = 0%, *Q* = 0.04, *p* = 0.8). These interventions did not reveal any improvement in postpartum hemorrhage among intervention recipients (SMD = −0.15, 95% CI −0.40 to 0.22). Similarly, no significant improvement was noted in maternal use of treatment services (SMD = −0.21, 95% CI −0.89 to 0.48, *I*^2^ = 76.55%). Other outcomes in this domain were reported in only one study each. Maternal admission to intensive care unit did not achieve statistical significance (SMD = −0.07, 95% CI −0.40 to 0.26, *n* = 1205). Sensitivity analyses did not reveal any significant changes in effects size for this outcome. No subgroup analyses were run for this outcome due to few studies reporting this outcome.

#### Breastfeeding practices

Initiation of breastfeeding was reported in only two studies, with no improvement estimated during pooled analyses (SMD = 0.05, 95% CI −0.06 to 0.46, *I*^2^ = 0%, *n* = 1574). Similar non-significance was also observed in exclusive breastfeeding practices (SMD = 0.01, 95% CI −0.11 to 0.13, *I*^2^ = 31.51%, *n* = 2438).

### Meta-analysis for child outcomes

A variety of child outcomes were reported in a total of six studies. Infant engagement was reported in one study (two trials), yielding non-significant effect sizes (SMD = 0.13, 95% CI −0.09 to 0.66, *I*^2^ = 0%, *n* = 302). Behavioral problems among infants were reported in two studies and did not show improvement among intervention recipients (SMD = 0.87, 95% CI 0.37–2.05, *I*^2^ = 0%). Outcome pertaining to APGAR score was reported in two studies, showing non-significance (OR 0.77, 95% CI 0.50–1.21, *I*^2^ = 0%). Similarly no improvement was observed in risk of low birth weight (OR 0.65, 95% CI 0.31–1.36, *I*^2^ = 78.45%, *n* = 2438); preterm birth (OR 0.17, 95% CI 0.01–6.34, *I*^2^ = 78.45%, *n* = 2438); perinatal mortality (OR 2.05, 95% CI 0.51–8.24, *I*^2^ = 0%, *n* = 2438); and missed immunizations (OR 0.77, 95% CI 0.50–1.21, *I*^2^ = 0%).

### Risk of bias

**Fig. 4.**
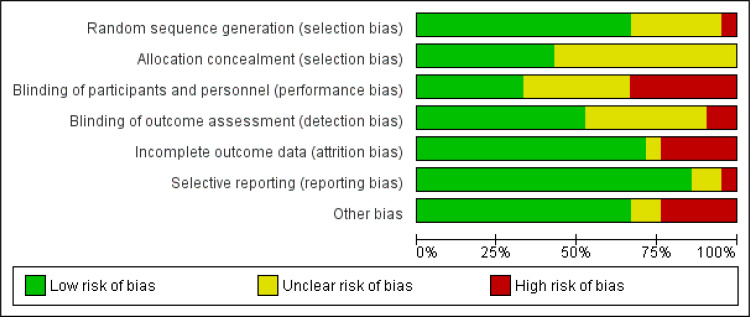
Risk of bias graph showing proportion of studies according to their risk of bias as per Cochrane tool.

A slightly higher proportion of studies (13 out of 21) presented with an overall higher risk of bias, with more than three matrices rated as high risk. Allocation concealment (*n* = 12) and blinding of participants and personnel (*n* = 14) and outcome assessors (*n* = 10) presented the highest risk of bias in included studies. These studies presented with the lowest risk in the domain of reporting bias (*n* = 3) (Fig. 4).

### Acceptability and feasibility

Data about the acceptability and feasibility of these interventions were reported in six studies (Cooper *et al*., 2015; Dimidjian *et al*., 2015; Brugha *et al*., 2016; Fisher *et al*., 2016; Greve *et al*., 2018; Duffecy *et al*., 2019). Overall, both the delivery agents and intervention recipients reported favorable attitudes toward these interventions. All these interventions were delivered by non-specialists except one which was delivered using online media (Duffecy *et al*., 2019). All of these studies were conducted in high-income countries in the UK (Brugha *et al*., 2016), the USA (Dimidjian *et al*., 2015; Duffecy *et al*., 2019), Australia (Fisher *et al*., 2016), and Norway (Greve *et al*., 2018). These studies had varying designs including cluster RCTs (*n* = 3) and feasibility or pilot RCTs (*n* = 3). The component of acceptability and feasibility of these studies was assessed using varying designs such as Likert scale type questionnaires (Cooper *et al*., 2015; Dimidjian *et al*., 2015; Fisher *et al*., 2016; Greve *et al*., 2018; Duffecy *et al*., 2019), and qualitative interviews (Brugha *et al*., 2016). In these studies, acceptability and feasibility were assessed among both the intervention providers and recipients. Intervention recipients mainly reported positive attitudes toward these interventions as evident by high compliance rates, positive attitudes toward delivery agents, and perceived usefulness and satisfaction toward the intervention.

### Attitude toward interventions

Cooper *et al*. assessed women's perceptions toward their intervention aimed at preventing postpartum depression by improving mother–infant relationship, using a Likert scale questionnaire (Cooper *et al*., 2015). Women were enrolled in two intervention arms delivered either by lay health visitors or trained NHS health visitors. Both types of interventions accrued positive responses by intervention recipients who felt better supported both emotionally and practically and helped facilitate fostering of a good mother–infant bond. In a similar vein, Fisher *et al*.'s intervention garnered positive reviews from intervention recipients where over 85% of the mothers and their partners reported that the psychoeducational intervention was useful and enjoyable and helped them develop infant caring skills and sharing work with their partners fairly (Fisher *et al*., 2016). High compliance rates were also reported by Greve *et al*., where completion rates of a psychotherapeutic intervention exceeded 95% (Greve *et al*., 2018).

Dimidjian *et al*. reported compliance rates, engagement, and satisfaction toward mindfulness-based cognitive-behavioral intervention program (Dimidjian *et al*., 2015). They reported high compliance rates (88%) among postpartum women and a high degree of satisfaction using a questionnaire. Around 83% of women enrolled in their intervention program reported an improvement in their coping skills toward intense emotions and an improved ability to recognize triggers and warnings (72%) and respond to them by engaging in positive activities (89%). Women at risk of postpartum depression receiving home visit interventions to improve mother–child relationships reported a high satisfaction toward the intervention. They found it particularly helpful in improving their understanding of infant behavioral cues and acknowledged ways in which their partners could support them in child-rearing. However, one of the mothers in this intervention felt that maternal and child health-related questions asked during intervention delivery were not age-appropriate. Positive sentiments toward the use of EPDS were evident in Brugha *et al*. for an intervention where women reported that its use improved their self-awareness toward depressive symptoms, and being offered help, goal setting, and homework set them on the right path in preventing postpartum depression (Brugha *et al*., 2016).

#### Attitude toward delivery agents

Fisher *et al*. used a Likert scale type questionnaire to assess the recipients' perceptions of the intervention (Fisher *et al*., 2016). Over 90% of intervention recipients and their partners agreed that facilitators were knowledgeable, well prepared, understood their needs, and were respectful toward their culture. Greve *et al*. reported that their intervention recipients found their delivery agents performing home visits to be trustworthy and easy to communicate with (Greve *et al*., 2018). Intervention recipients enrolled in Brugha *et al*., midwife-led cognitive-behavioral approaches were appreciative of the emotional care and reassurance provided to them (Brugha *et al*., 2016). Several women in this program cited the need for dedicated time for these listening visits- and felt rushed many times.

#### Perceptions of delivery agents

Brugha *et al*., in their midwife-led intervention program, was the only study reporting perspectives of delivery agents toward these interventions (Brugha *et al*., 2016). The midwives in this program particularly appreciated the focus on identifying depression, albeit concerned with the idea that time had to be allocated toward the assessment of postpartum depression and delivery of intervention. In addition, the slow process of the delivery of psychological therapies, and achieving remission was another critical aspect, which was found to be different in routine midwifery practices. Therefore, they felt the need to be allotted dedicated work hours for this, which was not usually the case even after assurances made by their managers.

### Cost-effectiveness

Dukhovny *et al*. presented a cost-effectiveness analysis for a volunteer-based program for the prevention of postpartum depression among high-risk Canadian women (Dukhovny *et al*., 2013). They reported that the mean cost per woman was $4497 in the peer support group and $3380 in the usual care group (difference of $1117, *p* < 0.0001). There was a 95% probability that the program would cost less than $20 196 per case of postpartum depression averted. Although this is a volunteer-based program, it resulted in a net cost to the health care system and society. However, this cost is within the range of other accepted interventions for this population. In another economic evaluation of a psychoeducational intervention, What Were We Thinking (WWWT) program in Australia, no differences in costs were revealed between the intervention recipients and their control counterparts (Ride *et al*., 2014). The incremental cost-effectiveness ratios were $A36 451 per quality-adjusted life-years (QALYs) gained and $A152 per percentage point reduction in the 30-day prevalence of depression, anxiety, and adjustment disorders. The estimate lies under the unofficial cost-effectiveness threshold of $A55 000 per QALY; however, there was considerable variability surrounding the results, with a 55% probability that WWWT would be considered cost-effective at that threshold.

### Quality of the evidence

Using the GRADE evidence guidelines (Guyatt, 2011), we chose six critical outcomes for quality assessment (Table 4). Overall, the quality of evidence across these outcomes ranged from low to high. The quality of evidence for the severity of depressive symptoms outcome was stepped down to moderate due to significant heterogeneity in reporting of these outcomes. Interventions aimed at perinatal depression significantly reduced the severity of perinatal depressive symptoms but no significant changes were observed in the reduction of rates of depressive disorders as per diagnostic criteria of DSM/ICD. However, for the severity of anxiety symptoms, there was high-quality evidence that these interventions yielded high effect sizes in favor of intervention recipients. For the outcome of rates of depression using SCID, DSM, or EPDS, the quality of evidence was high while for rates of generalized anxiety, it was judged to be low due to issues with inconsistency and risk of bias among studies. The evidence for relationship problems was judged to be moderate after stepping it down by one degree due to evidence of heterogeneity.

**Table 4.** GRADE evidence table showing certainty of evidence for six critical outcomes

Certainty assessment								No. of patients	Effect		Certainty	Importance
No. of studies	Study design	Risk of bias	Inconsistency	Indirectness	Imprecision	Other considerations	Psychological and psychosocial	care as usual	Relative (95% CI)	Absolute (95% CI)		
Severity of anxiety (assessed with: Psychometric scales)												
12	Randomized trials	Not serious^a^	Serious^b^	Not serious	Not serious	None	942	922	–	SMD **0.59 s.d. lower,** (0.95 lower to 0.23 lower)	⨁⨁⨁, Moderate	Critical
Rates of depressive disorders (assessed with: DSM/SCID/EPDS)												
7	Randomized trials	Not serious^a^	Not serious	Not serious	Not serious	None	167/1719 (9.7%)	204/1750 (11.7%)	OR 0.859 (0.650–1.130)	15 fewer per 1000, (from 38 fewer to 13 more)	⨁⨁⨁⨁, high	Critical
Severity of anxiety (assessed with: Psychometric scales)												
3	Randomized trials	Not serious	Serious^b^	Not serious	Not serious	Very strong association	229	203	–	SMD **1.53 s.d. lower,** (2.22 lower to 0.65 lower)	⨁⨁⨁⨁, high	Critical
Rate of generalized anxiety (assessed with: GAD-7/DSM)												
2	Randomized trials	Serious^a^	Serious^b^	Not serious	Not serious	None	−/251	−/248	OR 1.22 (0.74–2.01)	0 fewer per 1000, (from 0 fewer to 0 fewer)	⨁⨁, low	Critical
Utilization of health services												
2	Randomized trials	Serious^a^	Not serious	Not serious	Not serious	None	443	490	–	SMD **0.006 s.d. lower,** (0.12 lower to 0.13 higher)	⨁⨁⨁, moderate	Critical
Relationship problems (assessed with: Psychometric scales)												
4	Randomized trials	Not serious	Serious^b^	Not serious	Not serious	None	582	576	–	SMD **0.26 s.d. lower,** (0.48 lower to 0.04 lower)	⨁⨁⨁, moderate	Critical

CI, confidence interval; SMD, standardized mean difference; OR, odds ratio.

a A high proportion of studies reporting this outcome had a higher risk of bias.

b Substantial heterogeneity.

## Discussion

There is good quality evidence that psychological and psychosocial interventions delivered during the antenatal period prevent perinatal anxiety and depression. These interventions are found to be feasible and acceptable in different settings and cultures. In addition to preventing perinatal anxiety and depression, these also improve treatment-seeking attitudes and psychosocial functioning. However, the evidence for the cost-effectiveness of these interventions is sparse.

Our findings are corroborated by previous evidence on the effectiveness of preventive interventions (Dennis and Dowswell, 2014; Curry *et al*., 2019). Dennis and Dowswell (2014) reported a weak to moderate strength effect size for the reduction in symptoms and clinical diagnosis for postpartum depression. The review by the US Preventive Services Taskforce (USPSTF), reported similar findings for interventions conducted in primary care settings in high-income countries (Curry *et al*., 2019). Based on their review, the USPSTF recommended clinicians provide or refer pregnant and postpartum persons who are at an increased risk of perinatal depression to counseling interventions. The present analyses thus, build on the aforementioned reports by providing the latest evidence published globally.

For perinatal anxiety, however, the evidence must be interpreted with caution. Although these interventions show a good effect size in the reduction of symptoms of anxiety, the three studies reporting this outcome used the self-reported Spielberger state-trait anxiety inventory. Thus, this evidence may not be based on DSM and ICD criteria of diagnoses. Only three of the studies included in this evidence base targeted symptoms of anxiety as a primary outcome, however, these interventions did not employ therapeutic strategies specific to any psychological domain such as CBT or IPT. These were based on either psychoeducational principles or midwife-led care (Gu *et al*., 2013; Fisher *et al*., 2016; Sanaati *et al*., 2018).

The effectiveness of psychological and psychosocial interventions varies according to the timing of delivery of the intervention. As per our subgroup analyses, interventions should ideally be started in the antenatal period. The recent trials with interventions either delivered partly or wholly during the postpartum period were not found to be effective. However, it was observed that these interventions were based on varying theoretical backgrounds, principles, and content. And importantly, most of the trials in the latter set of studies tested psychosocial interventions only.

From the perspective of health systems, we found that these interventions do work when integrated with routine healthcare settings (Kenyon *et al*., 2016). Therefore, it is recommended that the core packages of mental health services (from prevention to management) are integrated into routine antenatal and postnatal care. For countries with developing economies, however, this may not be economically feasible. For such settings, inspiration could be taken from the World Health Organization's Mental Health Gap Action Programme (mhGAP) (World Health Organization, 2022). This could include the implementation of innovative strategies such as task-sharing by health workers or peers, training programs delivered electronically, or use of health applications, as well as establishment of effective referral mechanisms (Rahman *et al*., 2008; Atif *et al*., 2019). The Thinking Healthy Programme for perinatal depression is a task-shifting, clinically and cost-effective intervention for perinatal depression, however, it has not yet been tested for prevention of either perinatal anxiety or depression (Rahman *et al*., 2008).

### Strengths and limitations

The included evidence base lacked information about the implementation of these interventions. We could not find large-scale evaluation or feasibility studies reporting important implementation indicators such as training, supervision, and compensation of delivery agents. In addition, there was a lack of effort in developing standardized manuals of these interventions. These gaps in evidence severely impede efforts for their large-scale implementation in health systems. These also impede efforts for reproducibility and cross-cultural adaptation. Future studies should address the important implementation aspects of integration of these interventions into maternal and child health services, as well as planning for financial aspects; training and supervision; monitoring, and evaluation.

We found several gaps in prevention research for perinatal anxiety and depression. Evidence from low- and middle-income countries and rural settings was lacking in this systematic review. Most of the research has been conducted in the context of high-income countries. None of the interventions reported their effectiveness among refugees, migrants, and internally displaced perinatal women. There was only one intervention program designed for teen pregnancies which are prevalent in many traditional cultures. Future interventions should consider patient involvement in the development or tailoring of the interventions to local needs. Only two of the interventions (Fisher *et al*., 2016; Sanaati *et al*., 2018) ensured participation by new fathers in these interventions. This is important to target relevant risk factors of maternal and child health (e.g. intimate partner violence and involvement of the father in parental care). None of the studies reported longer-term follow-ups. A lack of research was noted in the outcomes related to infant health, morbidity and mortality, and early childhood development, with only six studies reporting these outcomes. Therefore, more research is recommended to report the effectiveness of these interventions in improving child health. There was substantial heterogeneity in the quality of the studies included in the review, therefore, the results of this meta-analysis should be generalized with caution.

## Conclusion

Interventions aimed at the prevention of anxiety and depression significantly reduced the severity of perinatal depressive and anxiety symptoms. These interventions were also found to be acceptable and feasible in many settings. We found several gaps in prevention research for perinatal anxiety and depression, especially in the context of implementation research.

## Data Availability

All data associated with this manuscript have been provided in the main text.

## Supplementary material

For supplementary material accompanying this paper visit http://dx.doi.org/10.1017/gmh.2022.17. click here to view supplementary material

## References

[ref1] Ahmed Waqas AR, Zafar S, Tariq M and Meraj H (2020) Prevention of common mental disorders among women in the postpartum period: a systematic review and meta-analysis. *PROSPERO* CRD42020166542. 10.1017/gmh.2022.17 PMC9806961 36618726

[ref2] American Psychiatric Association (2013) Diagnostic and Statistical Manual of Mental Disorders. Washington, DC: American Psychiatric Association.

[ref3] Atif N, Bibi A, Nisar A, Zulfiqar S, Ahmed I, LeMasters K, Hagaman A, Sikander S, Maselko J and Rahman A (2019) Delivering maternal mental health through peer volunteers: a 5-year report. International Journal of Mental Health Systems 13, 62–62. 31534475 10.1186/s13033-019-0318-3 PMC6747744

[ref4] Atif M, Halaki M, Raynes-Greenow C and Chow CM (2021) Perinatal depression in Pakistan: a systematic review and meta-analysis. Birth 48, 149–163. 33580505 10.1111/birt.12535

[ref5] Barrera AZ, Wickham RE and Muñoz RF (2015) Online prevention of postpartum depression for Spanish- and English-speaking pregnant women: a pilot randomized controlled trial. Internet Interventions 2, 257–265. 26273567 10.1016/j.invent.2015.06.002 PMC4530522

[ref6] Borenstein M, Hedges LV, Higgins JPT and Rothstein HR (2021) Introduction to Meta-Analysis. Chichester, UK: John Wiley & Sons.

[ref7] Brugha TS, Smith J, Austin J, Bankart J, Patterson M, Lovett C, Morgan Z, Morrell CJ and Slade P (2016) Can community midwives prevent antenatal depression? An external pilot study to test the feasibility of a cluster randomized controlled universal prevention trial. Psychological Medicine 46, 345–356. 26482473 10.1017/S003329171500183X PMC4682479

[ref8] Chorpita BF, Daleiden EL and Weisz JR (2005) Identifying and selecting the common elements of evidence based interventions: a distillation and matching model. Mental Health Services Research 7, 5–20. 15832690 10.1007/s11020-005-1962-6

[ref9] Cooper PJ, De Pascalis L, Woolgar M, Romaniuk H and Murray L (2015) Attempting to prevent postnatal depression by targeting the mother–infant relationship: a randomised controlled trial. Primary Health Care Research & Development 16, 383–397. 25381790 10.1017/S1463423614000401

[ref10] Curry SJ, Krist AH, Owens DK, Barry MJ, Caughey AB, Davidson KW, Doubeni CA, Epling JW, Grossman DC, Kemper AR, Kubik M, Landefeld CS, Mangione CM, Silverstein M, Simon MA, Tseng C-W and Wong JB (2019) Interventions to prevent perinatal depression. JAMA, 321, 580. 30747971 10.1001/jama.2019.0007

[ref11] Dennis C-L and Dowswell T (2014) Psychosocial and psychological interventions for preventing postpartum depression. Cochrane Database of Systematic Reviews 15(3), 231–233. 10.1002/14651858.CD001134.pub3 PMC11936315 23450532

[ref12] Dennis CL and Hodnett E (2007) Psychosocial and psychological interventions for treating postpartum depression. Cochrane Database of Systematic Reviews 2007(4), Art. No.: CD006116. doi: 10.1002/14651858.CD006116.pub2 17943888

[ref13] Dikmen-Yildiz P, Ayers S and Phillips L (2017) Depression, anxiety, PTSD and comorbidity in perinatal women in Turkey: a longitudinal population-based study. Midwifery 55, 29–37. 28917088 10.1016/j.midw.2017.09.001

[ref14] Dimidjian S, Goodman SH, Felder JN, Gallop R, Brown AP and Beck A (2015) An open trial of mindfulness-based cognitive therapy for the prevention of perinatal depressive relapse/recurrence. Archives of Women's Mental Health 18, 85–94. 10.1007/s00737-014-0468-x 25298253

[ref15] Dubber S, Reck C, Müller M and Gawlik S (2014) Postpartum bonding: the role of perinatal depression, anxiety and maternal–fetal bonding during pregnancy. Archives of Women's Mental Health 18, 187–195. 10.1007/s00737-014-0445-4 25088531

[ref16] Duffecy J, Grekin R, Hinkel H, Gallivan N, Nelson G and O'Hara MW (2019) A group-based online intervention to prevent postpartum depression (Sunnyside): feasibility randomized controlled trial. JMIR Mental Health 6, e10778–e10778. 31140443 10.2196/10778 PMC6707575

[ref17] Dukhovny D, Hodnett E, Weston J, Stewart D, Mao W, Zupancic J and Dennis C-L (2013) Prospective economic evaluation of a peer support intervention for prevention of postpartum depression among high-risk women in Ontario, Canada. American Journal of Perinatology 30, 631–642. 23283805 10.1055/s-0032-1331029

[ref18] Fathi-Ashtiani A, Ahmadi A, Ghobari-Bonab B, Azizi MP and Saheb-Alzamani SM (2015) Randomized trial of psychological interventions to preventing postpartum depression among Iranian first-time mothers. International Journal of Preventive Medicine 6, 109–109. 26682030 10.4103/2008-7802.169078 PMC4671165

[ref19] Fisher J, Cabral de Mello M, Patel V, Rahman A, Tran T, Holton S and Holmes W (2012) Prevalence and determinants of common perinatal mental disorders in women in low- and lower-middle-income countries: a systematic review. Bulletin of the World Health Organization 90, 139G–149G. 10.2471/BLT.11.091850 PMC3302553 22423165

[ref20] Fisher J, Rowe H, Wynter K, Tran T, Lorgelly P, Amir LH, Proimos J, Ranasinha S, Hiscock H, Bayer J and Cann W (2016) Gender-informed, psychoeducational programme for couples to prevent postnatal common mental disorders among primiparous women: cluster randomised controlled trial. BMJ Open 6, e009396–e009396. 10.1136/bmjopen-2015-009396 PMC4785308 26951210

[ref21] Fonseca A, Monteiro F, Alves S, Gorayeb R and Canavarro MC (2019) Be a mom, a web-based intervention to prevent postpartum depression: the enhancement of self-regulatory skills and its association with postpartum depressive symptoms. Frontiers in Psychology 10, 265–265. 30873060 10.3389/fpsyg.2019.00265 PMC6401984

[ref22] Gelaye B, Rondon MB, Araya R and Williams MA (2016) Epidemiology of maternal depression, risk factors, and child outcomes in low-income and middle-income countries. The Lancet, Psychiatry 3, 973–982. 27650773 10.1016/S2215-0366(16)30284-X PMC5155709

[ref23] Greve RA, Braarud HC, Skotheim S and Slinning K (2018) Feasibility and acceptability of an early home visit intervention aimed at supporting a positive mother–infant relationship for mothers at risk of postpartum depression. Scandinavian Journal of Caring Sciences 32, 1437–1446. 30011074 10.1111/scs.12589

[ref24] Gu C, Wu X, Ding Y, Zhu X and Zhang Z (2013) The effectiveness of a Chinese midwives’ antenatal clinic service on childbirth outcomes for primipare: a randomised controlled trial. International Journal of Nursing Studies 50, 1689–1697. 23735597 10.1016/j.ijnurstu.2013.05.001

[ref25] Guyatt GOA, Akl EA, Kunz R, Vist G, Brozek J, Norris S, Falck-Ytter Y, Glasziou P, DeBeer H and Jaeschke R (2011) GRADE guidelines: 1. Introduction – GRADE evidence profiles and summary of findings tables. Journal of Clinical Epidemiology 64, 383–394. 21195583 10.1016/j.jclinepi.2010.04.026

[ref26] Hantsoo L, Criniti S, Khan A, Moseley M, Kincler N, Faherty LJ, Epperson CN and Bennett IM (2018) A mobile application for monitoring and management of depressed mood in a vulnerable pregnant population. Psychiatric Services 69, 104–107. 29032705 10.1176/appi.ps.201600582 PMC5750085

[ref27] Higgins J, Thomas J, Chandler J, Cumpston M, Li T, Page M and Welch V (2019) Cochrane Handbook for Systematic Reviews of Interventions. Chichester, UK: John Wiley & Sons.

[ref28] Howard LM, Megnin-Viggars O, Symington I and Pilling S (2014) Antenatal and postnatal mental health: summary of updated NICE guidance. BMJ 349, g7394–g7394. 25523903 10.1136/bmj.g7394

[ref29] Howell EA, Bodnar-Deren S, Balbierz A, Loudon H, Mora PA, Zlotnick C, Wang J and Leventhal H (2014) An intervention to reduce postpartum depressive symptoms: a randomized controlled trial. Archives of Women's Mental Health 17, 57–63. 10.1007/s00737-013-0381-8 PMC3947932 24019052

[ref30] Jesse DE, Gaynes BN, Feldhousen EB, Newton ER, Bunch S and Hollon SD (2015) Performance of a culturally tailored cognitive-behavioral intervention integrated in a public health setting to reduce risk of antepartum depression: a randomized controlled trial. Journal of Midwifery & Women's Health 60, 578–592. 10.1111/jmwh.12308 PMC4775081 26261095

[ref31] Kaaya SF, Blander J, Antelman G, Cyprian F, Emmons KM, Matsumoto K, Chopyak E, Levine M and Fawzi MCS (2013) Randomized controlled trial evaluating the effect of an interactive group counseling intervention for HIV-positive women on prenatal depression and disclosure of HIV status. AIDS Care 25, 854–862. 23383726 10.1080/09540121.2013.763891

[ref32] Kenyon S, Jolly K, Hemming K, Hope L, Blissett J, Dann S-A, Lilford R and MacArthur C (2016) Lay support for pregnant women with social risk: a randomised controlled trial. BMJ Open 6, e009203–e009203. 10.1136/bmjopen-2015-009203 PMC4785315 26936901

[ref33] Krusche A, Dymond M, Murphy SE and Crane C (2018) Mindfulness for pregnancy: a randomised controlled study of online mindfulness during pregnancy. Midwifery 65, 51–57. 30099285 10.1016/j.midw.2018.07.005

[ref34] Luca DL, Margiotta C, Staatz C, Garlow E, Christensen A and Zivin K (2020) Financial toll of untreated perinatal mood and anxiety disorders among 2017 births in the United States. American Journal of Public Health 110, 888–896. 32298167 10.2105/AJPH.2020.305619 PMC7204436

[ref35] Maimburg RD and Væth M (2015) Postpartum depression among first-time mothers – results from a parallel randomised trial. Sexual & Reproductive Healthcare 6, 95–100. 25998877 10.1016/j.srhc.2015.01.003

[ref36] Morrell C, Warner R, Slade P, Dixon S, Walters S, Paley G and Brugha T (2009) Psychological interventions for postnatal depression: cluster randomised trial and economic evaluation. The PoNDER trial. Health Technology Assessment 13, 1–176. 10.3310/hta13300 19555590

[ref37] Moshki M, Baloochi Beydokhti T and Cheravi K (2014) The effect of educational intervention on prevention of postpartum depression: an application of health locus of control. Journal of Clinical Nursing 23(15–16), 2256–2263. 24329943 10.1111/jocn.12505

[ref38] Muzik M, Thelen K and Rosenblum KL (2011) Perinatal depression: detection and treatment. Neuropsychiatry 1, 179–195.

[ref39] National Institute for Health and Care Excellence (2014) *Antenatal and postnatal mental health: Clinical management and service guidance (update) (Clinical Guideline 192)*. 31990493

[ref40] Ortiz Collado MA, Saez M, Favrod J and Hatem M (2014) Antenatal psychosomatic programming to reduce postpartum depression risk and improve childbirth outcomes: a randomized controlled trial in Spain and France. BMC Pregnancy and Childbirth 14, 22–22. 24422605 10.1186/1471-2393-14-22 PMC3898772

[ref41] Ouzzani M, Hammady H, Fedorowicz Z and Elmagarmid A (2016) Rayyan – a web and mobile app for systematic reviews. Systematic Reviews 5, 210–210. 27919275 10.1186/s13643-016-0384-4 PMC5139140

[ref42] Phipps MG, Raker CA, Ware CF and Zlotnick C (2013) Randomized controlled trial to prevent postpartum depression in adolescent mothers. American Journal of Obstetrics and Gynecology 208, 192.e1–192.e1926. 10.1016/j.ajog.2012.12.036 PMC4386618 23313720

[ref43] Rahman A, Malik A, Sikander S, Roberts C and Creed F (2008) Cognitive behaviour therapy-based intervention by community health workers for mothers with depression and their infants in rural Pakistan: a cluster-randomised controlled trial. The Lancet 372, 902–909. 10.1016/S0140-6736(08)61400-2 PMC2603063 18790313

[ref44] Ride J, Rowe H, Wynter K, Fisher J and Lorgelly P (2014) Protocol for economic evaluation alongside a cluster-randomised controlled trial of a psychoeducational intervention for the primary prevention of postnatal mental health problems in first-time mothers. BMJ Open 4, e006226–e006226. 10.1136/bmjopen-2014-006226 PMC4187457 25280810

[ref45] Sanaati F, Charandabi SM-A, Eslamlo HF and Mirghafourvand M (2018) A randomized controlled trial on the effect of lifestyle education for Iranian women and their husbands on post-partum anxiety and depression. Health Education Research 33, 416–428. 30107480 10.1093/her/cyy026

[ref46] Singla DR, Kohrt BA, Murray LK, Anand A, Chorpita BF and Patel V (2017) Psychological treatments for the world: lessons from low- and middle-income countries. Annual Review of Clinical Psychology 13, 149–181. 10.1146/annurev-clinpsy-032816-045217 PMC5506549 28482687

[ref47] Stein A, Pearson RM, Goodman SH, Rapa E, Rahman A, McCallum M, Howard LM and Pariante CM (2014) Effects of perinatal mental disorders on the fetus and child. The Lancet 384, 1800–1819. 10.1016/S0140-6736(14)61277-0 25455250

[ref48] Tandon SD, Leis JA, Mendelson T, Perry DF and Kemp K (2014) Six-month outcomes from a randomized controlled trial to prevent perinatal depression in low-income home visiting clients. Maternal and Child Health Journal 18(4), 873–881. 23793487 10.1007/s10995-013-1313-y PMC3871944

[ref49] Waqas A, Raza N, Lodhi HW, Muhammad Z, Jamal M and Rehman A (2015) Psychosocial factors of antenatal anxiety and depression in Pakistan: is social support a mediator?. PloS one 10(1), e0116510. 25629925 10.1371/journal.pone.0116510 PMC4309576

[ref50] Waqas A, Elhady M, Surya Dila KA, Kaboub F, Van Trinh L, Nhien CH, Al-Husseini MJ, Kamel MG, Elshafay A, Nhi HY, Hirayama K and Huy NT (2018) Association between maternal depression and risk of infant diarrhea: a systematic review and meta-analysis. Public Health 159, 78–88. 29627116 10.1016/j.puhe.2018.01.036

[ref51] World Health Organization (1992) *The ICD-10 classification of mental and behavioural disorders: clinical descriptions and diagnostic guidelines*.

[ref52] World Health Organization (2022) *mhGAP Intervention Guide Mental Health Gap Action Programme (Version 2.0) for mental, neurological and substance use disorders in non-specialized health settings*. 23741783

[ref53] Zafar S, Sikander S, Haq Z, Hill Z, Lingam R, Skordis-Worrall J, Hafeez A, Kirkwood B and Rahman A (2014) Integrating maternal psychosocial well-being into a child-development intervention: the five-pillars approach. Annals of the New York Academy of Sciences 1308, 107–117. 24571213 10.1111/nyas.12339

[ref54] Zlotnick C, Miller IW, Pearlstein T, Howard M and Sweeney P (2006) A preventive intervention for pregnant women on public assistance at risk for postpartum depression. American Journal of Psychiatry 163(8), 1443–1445. 16877662 10.1176/appi.ajp.163.8.1443 PMC4387544

