# Supplementary material for: Prevention of common mental disorders among women in the perinatal period: a critical mixed-methods review and meta-analysis
Source: Glob Ment Health (Camb). 2022 Mar 23;9:157–72. doi: 10.1017/gmh.2022.17 (PMC9806961; doi:10.1017/gmh.2022.17)
Supplement: Supplementary file 1 [file S2054425122000176sup.zip › S2054425122000176sup001.docx]

Supplementary figure 1: Effectiveness of psychological and psychosocial interventions in depressive symptoms

Supplementary figure 2: Effectiveness of psychological and psychosocial interventions in depressive disorder

Supplementary figure 3: Effectiveness of psychological and psychosocial interventions in anxiety symptoms

Supplementary figure 4: Effectiveness of psychological and psychosocial interventions in anxiety disorder

Supplementary figure 5: Effectiveness of psychological and psychosocial interventions in self-esteem

Supplementary figure 6: Effectiveness of psychological and psychosocial interventions in marital problems

Supplementary figure 7: Meta-regression plot for association between depression severity and number of sessions

Supplementary figure 8: Meta-regression plot for association between depression severity and duration of sessions

Supplementary figure 9: Funnel plot for visualizing publication bias in depressive symptoms
