# Supplementary material for: Prevention of common mental disorders among women in the perinatal period: a critical mixed-methods review and meta-analysis
Source: Glob Ment Health (Camb). 2022 Mar 23;9:157–72. doi: 10.1017/gmh.2022.17 (PMC9806961; doi:10.1017/gmh.2022.17)
Supplement: Supplementary file 1 [file S2054425122000176sup.zip › S2054425122000176sup002.docx]

**Supplementary table 1:** Demographic characteristics of participants included in intervention

| **Author, year** | **Study design** | **Age range of mothers** | **Geographical scope** | **Setting of intervention** | **Tools used for mental health assessment** | **Inclusion criteria: Mothers** |
| --- | --- | --- | --- | --- | --- | --- |
| Zlotnick et al, 2016 | RCT/efficacy trial | 22.7 (4.4) years | 999 | Hospital | Rate of postpartum depression, Beck Depression Inventory, Range of Impaired Functioning Tool, Depression Module of the Longitudinal Interval Followup Evaluation | Inclusion criteria for the study were: 1) pregnant status, 2) 18 years or older, 3) between 20 and 35 weeks gestation, 4) received public assistance, 5) English-speaking, 6) attended an urban, pre-natal medical clinic in the Northeast, and 7) a score of 27 or more on the Cooper Survey Questionnaire (First et al., 2002) (CSQ; see Procedures), which is the empirically derived threshold for high-risk status |
| Phipps et al, 2013 | RCT | Intervention=16 (13-18), Control=16(14-18) | Urban | Hospital | 999 | Participants were eligible if they were #17 years old when they con-ceived their pregnancy and were 25 weeks gestational age at their first prena-tal visit. |
| Hantsoo et al, 2017 | RCT | Control group=26.36 (4.9), Intervention group= 26.56 (6.2) | Urban | Prenatal clinic within an academic medical center | Depression=PHQ-9, Anxiety=GAD-7 | Women 18 years old and 32 weeks gestation or less, per electronic health record (EHR), were approached by a research coordinator at a routine prenatal appointment. Enrollment was offered to English-speaking women with depressive symptoms ($5 on the Patient Health Questionnaire–9 [PHQ-9]) who owned a smartphone with an iOS or Android operating system |
| Howell et al, 2014 | RCT | Intervention group=33 (6), control group=32(5) | Urban | Large tertiary inner city hospital located in East Harlem, New York City | Depressive symptoms: Edinburgh Postnatal Depression Scale (EPDS) | Age ≥18 years |
| Jesse et al, 2015 | RCT | 25.05 (5.49) | Rural | Hospital | Postpartum Depression=Edinburgh Postnatal Depression Scale (EPDS),42 the Beck Depression Inventory-II,41 and MINI 6.0 | Women who were 18 years or older; between 6 and 30 weeks pregnant, enrolled at the local health department (LHD) or an affiliated regional perinatal center; self-identified as African American, Caucasian, or Hispanic; able to read at a fourth-grade level; scored 4 or higher on the EPDS;39 and enrolled in Medicaid or were low income based on Special Supplemental Nutrition Program for Women, |
| Kenyon et al, 2017 | Prospective, pragmatic, individually randomised controlled trial. | Intervention=21.8 (19.0, 25.5), Control group=21.5 (18.8, 24.6) | 999 | 3 Maternity Trusts in West Midlands, UK. | Maternal depression: Edinburgh Postnatal Depression Scale17 (EPDS) | 1. Nulliparous women < 28 weeks gestation 2. Assessed for specified social risk through systematic assessment. |
| Tandon et al, 2014 | RCT | 24.1 ( 6.1) | 999 | Home | Depression=Beck Depression Inventor (BDI-II) | Women with elevated depressive symptoms (CES-D C16) and/or a lifetime depressive episode but who were not currently exhibiting a depressive episode were eligible for study participation. |
| Sanaati et al, 2018 | RCT | 27.8 (5.0) | Urban | Hospital | Postpartum Depression=EPDS | 8 weeks, an uncomplicated singleton pregnancy, an Edinburgh Postnatal Depression Inventory score of lower than 12, ﬁrst or secondpregnancy, a medical record in health centers of Bukan (a city in West Azerbaijan Province, Iran), at least a secondary education, not participating in other similar studies, willing to participate in the study, having a contact number for follow-ups, willingness of the mother and father to attend the classes and receive telephone counseling |
| Gu et al, 2013 | Two-group randomised controlled trial | 29.01 (2.55) | 999 | Hospital | Childbirth outcomes, women’s psychological state and satisfaction /State-Trait Anxiety Inventory | inclusion criteria: (1) Mandarin-speaking: able to speak, read and write in Chinese; (2) 29–30 weeks gestation at recruit-ment; (3) low risk at recruitment in absence of medical or obstetrical complications; (4) singleton pregnanc |
| Maimburg et al, 2015 | RCT | Intervention= 28.9(3.7), Control Group= 29.7 (3.7) | Urban | Hospital | Postpartum Depression= Edinburgh Postnatal Depression Scale | The inclusion criteria were pregnant between 10+0 to 21+6 days of gestation, nullipara, registered at the Aarhus Midwifery Clinic, older than 18 years of age at enrolment, singleton pregnancy, and ability to speak and understand Danish. |
| Collado et al, 2014 | RCT | Intervention=29 (5.2), Control group=28.5 (6.2) | Urban | Hospital | 1) the depressive symptoms: EPDS scale; 2) amount of social support receiveL Functional Social Support Questionnaire of Broadhead et al 3) stressful events, based on Holmes and Rahe, 1967; and 4) relationship with the partner using Spanier’s dyadic adjustment scale DAS, 1976 | The participants were pregnant women and their partners considered to be at psychosocial risk via three factors: socioeconomic status (low-paid jobs, unemployed, with or without subsidy), low social support (migrants or those living isolated), and the risk of PPD (validated interview). Couples were eligible to take part in the study if (1) they were identified at middle or low socio economic status (based on income, occupational category and type of employment contract, an indicator of job security), and (2) the women met these individual criteria: a) pregnancy ≤ 20 weeks; b) a moderate to high risk of PPD; c) no more than two children; d) no organic serious physical pathology; e) no psychiatric diagnosis; f) no alcohol or illicit substance abuse, and (g) understand the language of the study. |
| Cooper et al, 2015 | RCT | Intervention=27.94 (5.4), Control group=28.66 (6) | 999 | home based session | Structured Clinical Interview for DSM-IV Axis I Disorders, Postparum Depression= Edinburgh Postnatal Depression Scale (EPDS) | single pregnancy, stable residence in the area, English the home language), women with score of more than 15 |
| Fathi-Ashtiani, 2015 | quasi-experimental study | 25.8 ( 3.7) | 999 | 999 | Postpartum Depression=Edinburgh postnatal depression scale, Beck depression inventory, Postpartum Anxiety=Beck anxiety inventory | Participants aged 18–32 years; had a single birth; an uncomplicated pregnancy, ability to read and write |
| Barrera et al, 2015 | Pilot RCT | 30.19 years (SD = 5.57) | 999 | Fully-automated self-help Internet intervention | Center for Epidemiologic Studies-Depression Scale (CES-D) | Female, pregnant, 18 years or older, and interested in the study website for personal use |
| Fonseca et al, 2019 | Pilot RCT | Intervention group=32.22 (4.36), Control group=32.94 (5.24) | Mixed: Both Urban and Rurtal | Community | Edinburgh Postpartum Depression Scale=Postpartum depressive symptoms | adult ( 18 years), in the early postpartum period (up to 3 months postpartum), and presented risk factors for PPD, Have access to a computer/tablet/smartphone and internet access at home, the ability to read and speak Portuguese and to be a Portuguese resident |
| Krusche et al , 2018 | Pilot RCT | Range=22-40 years, Mean age=32.7 years, | 999 | Online Intervention | The Perceived Stress Scale (PSS), The General Anxiety Disorder-7 (GAD-7), The Edinburgh Postnatal Depression Scale (EPDS), The Tilburg Pregnancy Distress Scale (TPDS), The Oxford Worries about Labour Scale (OWLS) | Expecting mothers Age 18 or above |
| Fisher et al, 2016 | Cluster RCT | Intervention group=31.8 (5.2), Control group=32.0 (5.1) | 999 | Was implenented in standardard setting | DSM IV, Diagnostic and Statistical Manual of Mental Disorders, fourth Edition; GAD, Generalised Anxiety Disorder; PHQ, Patient Health Questionnaire; | Primiparous women with adequate English fluency residing in participating local government areas who have given birth in the previous two weeks and are receiving care in a trial maternal and child health centre. Both the mother and father will be invited and encouraged to attend, however in situations where the father is unable or unwilling to attend, mothers will be encouraged to attend with a nominated support person, for example, their mother, friend or grandmother, but if this is not possible, women will be welcome to attend alone. Parents in same sex relationships are invited and welcome to attend. |
| Duffecy et al, 2019 | Feasibility Randomized Controlled Trial | 30.5 years (SD 4.05) | Urban | Online intervention | The Hamilton Depression Rating Scale (HDRS),The Inventory of Depression and Anxiety Symptoms (IDAS) ,The Patient Health Questionnaire (PHQ-8) | Participants 18 years of age or older, between 20-and 28-weeks gestation at the time of baseline assessment, had a score between 5 and 14 on the PHQ-8 screener (mild-moderate depressive symptoms), were able to read and speak English, and had access to the internet on any device. |
| Brugha et al, 2016 | Feasibility RCT | 999 | Mixed: Both Urban and Rurtal | Community | Edinburgh Perinatal Depression Scale’ State-Trait Anxiety Inventory, the Agnew Relationship Scale – short form,and Satisfaction with Life Scale | Edinburgh Perinatal Depression Scale’ State-Trait Anxiety Inventory, the Agnew Relationship Scale – short form,and Satisfaction with Life Scale |
| Moshki et al, 2015 | pre–post experimental design with a control group | Intervention=28(6.39), Control group=27.8 (5.29) | Urban | 999 | Postpartum Depression=Edinburgh Postnatal Depression Scale/Multidimensional health locus of control Scale ( | Study inclusion criteria were gestational age of 28–30 weeks, literacy, no history of depression or psychological disorders, healthy foetus conﬁrmed by ultrasound and residence in Gonabad |
| Dimidjian et al, 2016 | Pilot RCT | Intervention: 30.98 (4.08), Control group=28.72 (5.50) | 999 | Hospital | Structured Clinical Interview for DSM-IV-TR | pregnant women (a) up to 32 weeks gestation, (b) meeting criteria for prior major depressive disorder (MDD), (c) available for group intervention scheduled meetings, and (d) aged 18 years or older. |

**Supplementary table** 2: Description of interventions included in the review

| **Study ID** | **Intervention category** | **Content of the intervention** |
| --- | --- | --- |
| Barrera et al, 2015 | CBT | Introduction, mother-baby relationship, Identifying stressors, Balance life stressors with healthy mood management skills, Identify pleasant activities, Psychoeducation, thoughts-self talk, Contact beween mood and support, intergenerational way of thinking |
| Brugha et al, 2016 | CBT | CBA |
| Duffecy et al, 2019 | CBT | Your mood and pregnancy, worries about me and my baby, mood management, challenging your thinking, positive activity during pregnancy, physical activity during pregnancy, partner communication and support, body image and sex during pregnancy and the postpartum, Relationships with your Mother and Mother-in-Law, Challenges in Relationships with Friends and others, Monitoring Kick Counts and Other Pregnancy Anxiety, Anxiety and Parenthood, Relaxation, Employment Issues, During and After the Birth: How to Manage and Resources, Moving Forward and Conclusions |
| Fisher et al, 2016 | Psychoeducation | Three components including program theory and implementation, print material and fac-to-face seminar covering following areas: About Babies includes learning activities about infant temperament, crying and fussing, recognition of tired cues, sleep needs, establishing feed-play-sleep routines of daily care and safe, sustainable settling strategies: known collectively as ‘infant behaviour management’. About Parents includes learning activities about differences between how parenthood had been imagined and is being experienced; recal-ling the difficult and pleasing aspects of the baby’s birth; recognising, naming and renegotiating the unpaid workload fairly in non- confrontational ways; acknowledging the disenfranchised losses of parenthood as well as the gains; identifying experiences within parents’ families of origin that they wish to duplicate or to relinquish; and identifying gaps in support. |
| Fonseca et al, 2019 | CBT, acceptance and compassion based therapy | **Session 1:** Changes and reorganizations during transition to parenthood, Unrealistic expectations, Role idealization and Perfect motherhood, Emotional experience, Cognitive-emotional-behavioral link[] Session 2: Negative thoughts, Reducing the power of thoughts: questioning and defusion, Self-criticism and self-compassion [] Session 3: Values and commitment, Social network: How to identify support needs and ask for help;, Assertive communication: Dealing with family and friends [] Session 4: Changes in the couple relationship during the postpartum period, Assertive communication within the couple: negotiation and conflict resolution skills;, Sharing parenthood values and commitments [] Session 5: Identify PPD signs and symptoms, Professional help-seeking: treatment options and how to seek help, A continuing journey: planning for the future; |
| Hantsoo et al, 2017 | Psychoeducation/Mood monitoring | Mood monitoring,psychoeducation, physical activity |
| Howell et al, 2014 | Behavioral education | 2-step behavioral educational intervention. The in-hospital component and a call after 2 weeks |
| Jesse et al, 2015 | CBT | Session 1: Introductions, Symptoms of Depression in Pregnancy, Goal-setting, Distorted Thinking, and Pleasant Life Activities [] Session 2: Stress Reduction, Coping, Relaxing, Automatic Negative Thoughts, and Positive Affirmations [] Session 3: Relationship Evaluation and Goals, and Domestic Violence [] Session 4: Communication, Problem Solving, and Group Session with Invited Support Person [] Session 5: Caregiving, Grief, Healing from Loss, Spirituality, and Other Resources [] Session 6: Signs and Symptoms of Postpartum Depression, Role Transitions, and Saying Goodbye |
| Kenyon et al, 2017 | Psychosocial | Objectives were to encourage women to attend antenatal appointments, make healthy lifestyle choices, to provide social/emotional support, and help ensure benefits, housing difficulties and mental health problems were managed. In the postnatal period (to 6 weeks postpartum), POWs also provided breast feeding and advice about infant care. |
| Krusche et al, 2018 | Mindfulness | Introduction, Stepping out of Automatic Pilot, Reconnecting with Body & Breath, Working with Difficulties, Mindfulness in Daily Life, Going Forward |
| Dimidjian et al, 2016 | Mindfulness based Cognitive Therapy |  |
| Tandon at al, 2014 | CBT | Pleasant activities, thoughts, and contact with others. The activities and group discussion focused largely on introducing and practicing the use of core skills (e.g., strategies to reduce harmful thought patterns, ways to effectively ask for support) |
| Sanaati et al, 2018 | Lifestyle education | Anatomy and physiology of genital organs and preg- nancy, sleep hygiene, nutrition, pregnancy ex- ercises, sexual matters |
| Fathi-Asthiani et al, 2015 | CBT | negative thoughts were triggered, the person would expect negative and aversive event about him or herself, the world, and the future. The treatment consisted of training in self-monitoring, self-focused attention, relaxation, understanding of the problem, and setting up an alternative view of the problem, revising automatic thoughts, behavioral approach tasks, and exposure to worry cues. Other themes of the ECBSP included education on depressive and anxiety symptomatology, positive communication, realistic expectation about pregnancy, delivery, and parenting, recording their mood, pleasurable activities, and relaxation exercise. The patients received the homework to ensure transfer of skills from therapeutic contexts to daily life. |
| Gu et al, 2013 | Antenatal education | The midwife usually focussed on antenatal checkups, consultation, making birth plans, parent educa- tion, and collaborated with obstetricians and other health professionals as necessary. The midwife would be on call for the woman’s labour and birth except in designated circumstances such as annual leave; sick leave; having already worked more than 16h in a 24-h period; and having more than one woman in labour. |
| Moshki et al, 2014 | Education-based | anatomic and physi-ological changes, nutrition, common complications during pregnancy, mental health and communication skills, familiarisation with pregnancy stages, delivery and pain reducion methods, postpartum health, emotions and attitudes of women with special emphasis on components of HLC |
| Maimburg et al, 2015 | Antenatal education | The content of the birth module included lectures on and discussion of labour onset, the birth process, the father's role during birth, pain relief, birth interventions and fear of childbirth, and a film on giving birth. The newborn module included lectures on and discussions of care for the newborn, breastfeeding, childhood diseases, vaccinations, and equipment and safety measures for the child. The parent module included lectures on and discussion of transition to parenthood, maternity leave, sexual relations, conflicts in parental relationship, the role of grandparents, family and friends, and PPD. The PPD lecture included information on prevalence, prevention, symptoms (shared and different symptoms in men and women), and PPD treatment. |
| Collado et al, 2014 | Humanist | Developing a therapeutic alliance, normalizing antenatal somatic symptoms, developing alternative explanations for their sensations and experience, and connecting somatic symptoms to emotion. exercises for reasoning with somatic symptoms and childbirth model |
| Zlotnick et al, 2016 | IPT based | The content of the intervention focuses on managing role transitions with an emphasis on transition to motherhood, developing a support system, developing effective communication skills to manage relationship conflicts before and after the birth of their baby, goal setting, and psychosocial resources for new mothers |
| Phipps et al, 2013 | IPT based | development of effective communication skills to manage relationship conflicts before and after the birth of the baby, expectations about motherhood, stress management, “baby blues” vs depression, development of a support system, development of healthy relationships, goal setting, and psychosocial resources for new mothers |
| Cooper et al, 2015 | Supportive therapy, infant behavioral assessment based | The intervention comprised three principal elements. First, supportive counselling was provided. Second, specific strategies were employed to sensitise the mothers to their infants’ characteristics. inally, specific help was provided to the mothers in managing infant behavioural problems (sleeping, feeding, crying). |
